# Diagnostic Value of Spectral CT–Derived Extracellular Volume Fraction in the Early Detection of Sigmoid Colon Metastasis from Bladder Cancer: A Case Report

**DOI:** 10.70352/scrj.cr.26-0147

**Published:** 2026-06-20

**Authors:** Maolan Peng, Lu Shen, Deqing Song, Lei Wen, Yining Xiang, Lingling Song

**Affiliations:** 1 Department of Radiology, Affiliated Hospital of Guizhou Medical University, Guiyang, Guizhou, China; 2 Department of Pathology, Affiliated Hospital of Guizhou Medical University, Guiyang, Guizhou, China

**Keywords:** bladder cancer, spectral CT, extracellular volume, sigmoid colon metastasis

## Abstract

**INTRODUCTION:**

The occurrence of colorectal metastasis from bladder cancer is an uncommon phenomenon, and the early identification of metastatic foci poses a certain challenge.

**CASE PRESENTATION:**

A 77-year-old male patient who had been diagnosed with bladder carcinoma underwent radical cystectomy with an ileal conduit. Postoperative spectral contrast-enhanced CT revealed no definitive intra-abdominal metastatic lesions. Following a period of 1 month, the patient presented with abdominal pain and melena, and subsequent spectral contrast-enhanced CT revealed a metastatic lesion in the sigmoid colon and multiple hepatic metastases. A retrospective analysis of the initial postoperative extracellular volume fraction (ECV) images from spectral CT clearly delineated the metastatic lesion in the sigmoid colon, despite the absence of abnormalities on conventional images.

**CONCLUSIONS:**

This case report suggests that multiparametric spectral CT, particularly ECV imaging, may detect subtle tissue changes before apparent morphological changes, indicating its potential value in the recognition of early metastatic lesions. To our knowledge, this is the first reported case demonstrating the potential value of spectral CT–derived ECV imaging for the early detection of sigmoid colon metastasis from bladder cancer.

## Abbreviations

ECV
: extracellular volume fraction
GI
: gastrointestinal
Hct
: hematocrit
ROI
: region of interest
TURBT
: transurethral resection of the bladder tumor
VMI
: virtual monoenergetic images

## INTRODUCTION

Bladder cancer is one of the most common malignant tumors of the urinary system and ranks ninth worldwide in terms of incidence.^1)^ The metastasis rate was 29%, with common metastatic sites including lymph nodes (25.4%), bone (24.7%), urinary tract (23.5%), lung (19.4%), liver (18.1%). and brain (3.1%).^1)^ In contrast, colorectal metastases from bladder cancer are extremely rare and typically occur at advanced or recurrent stages, often indicating a poor prognosis.

According to the current guidelines of the American Urological Association, CT is recommended as the primary imaging modality for the diagnosis and postoperative surveillance of bladder cancer.^2)^ Conventional CT evaluates bladder tumors by morphological characteristics, attenuation values, and enhancement features. However, this technique has inherent limitations, particularly in the detection of early-stage, small metastatic lesions. Insufficient lesion-to-background contrast may further contribute to false-negative findings, thereby compromising diagnostic accuracy.^3)^ In recent years, spectral CT has emerged as an advanced multiparametric imaging technique that enables material decomposition and energy-resolved data acquisition. It enables the generation of VMI, iodine density maps, and ECV maps. These parameters enhance lesion-to-background contrast and provide quantitative metrics reflecting tumor perfusion and microenvironmental characteristics, thereby potentially enhancing the detection rate of lesions.^3–5)^

The present case report describes a missed diagnosis of sigmoid colon metastasis following bladder cancer surgery. Retrospective analysis of the initial postoperative abdominal spectral contrast-enhanced CT demonstrated that the ECV map indicated the sigmoid metastatic lesion, even when conventional CT images appeared normal. These findings suggest the potential role of spectral CT parameters in earlier recognition of metastatic lesions.

## CASE PRESENTATION

A 77-year-old male patient with a long history of tobacco smoking was diagnosed with high-grade invasive urothelial carcinoma of the bladder (pT1N0M0, stage I). The patient underwent TURBT with bladder-neck resection, followed by intravesical instillation of doxorubicin for antitumor therapy. Given the aggressive nature of the tumor, radical cystectomy with ileal conduit diversion was performed, and the patient received targeted immunotherapy with vidicitumumab and toripalimab. One month post-operation, the patient underwent postoperative abdominal spectral contrast-enhanced CT. No definitive evidence of intra-abdominal metastatic disease was identified on conventional images (**Fig. 1A**). Two months post-operation, the patient was readmitted to the hospital with paroxysmal abdominal pain and intermittent melena. Colonoscopy revealed an elevated lesion in the sigmoid colon (**Fig. 1B**). A subsequent abdominal spectral contrast-enhanced CT examination confirmed the presence of a metastatic lesion in the sigmoid colon (**Fig. 1C**) and multiple hepatic metastases (**Fig. 1D**).

**Fig. 1:**
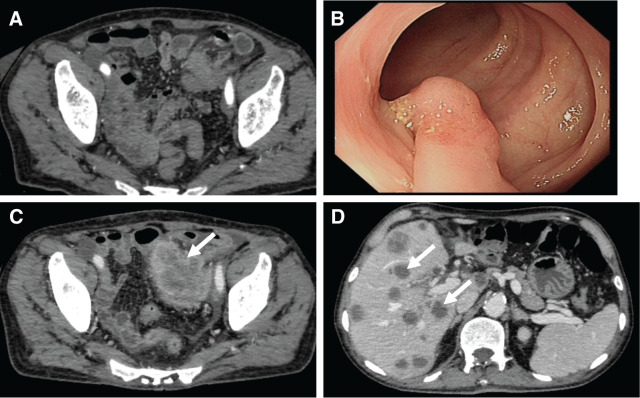
Abdominal spectral contrast-enhanced CT and colonoscopy findings. (**A**) No metastasis was detected 1 month post-operation. (**B**) Two months later, colonoscopy revealed an elevated sigmoid colon lesion. (**C**, **D**) Spectral CT 2 months later demonstrated apparent metastatic lesions in the sigmoid colon and liver (arrows).

On the second abdominal spectral CT scan, the 50-keV VMI demonstrated a heterogeneously enhancing mass located slightly to the left of the pelvic midline. The lesion exhibited markedly increased contrast relative to the adjacent normal bowel wall, and the tumor–bowel interface was more clearly delineated (**Fig. 2A**). The iodine-no-water image showed pronounced iodine accumulation within the lesion (**Fig. 2B**). On the ECV map, the lesion was clearly visualized and exhibited a markedly elevated ECV value of 86.9% (**Fig. 2C**). ECV was measured on the equilibrium-phase maps generated by the spectral CT workstation (Philips IntelliSpace Portal; Philips, Amsterdam, the Netherlands). A circular ROI (area 39.73 mm^2^) was placed within the lesion while avoiding vessels, calcifications, necrosis, and tissue margins to minimize partial volume effects. The Hct value (0.21) used for calculation was obtained from a contemporaneous peripheral blood test. The workstation automatically calculated the mean ECV value using the formula: ECV (%) = (1−Hct) × (iodine density of tissue/iodine density of blood pool) × 100. A reference ROI was placed in the abdominal aorta to obtain the iodine concentration of the blood pool. The slope value of the spectral attenuation curve derived from the sigmoid colon lesion and hepatic metastases exhibited analogous slope values (**Fig. 2D**), suggesting a shared pathological origin from the primary bladder carcinoma.

**Fig. 2:**
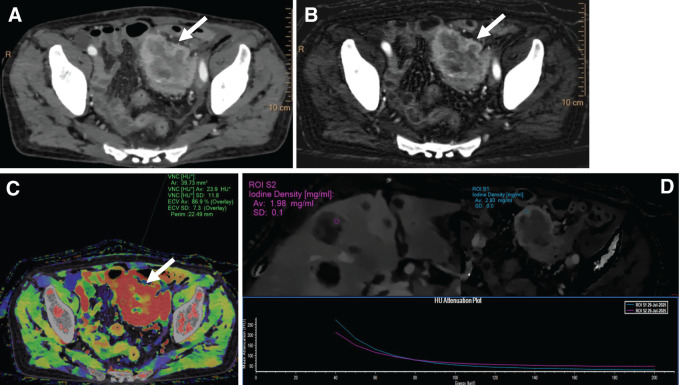
Spectral CT images 2 months post-operation. (**A**) Fifty-keV monoenergetic image (arrow) and (**B**) iodine-no-water image (arrow). (**C**) ECV map (arrow). (**D**) Spectral attenuation curves showed that the S1 and S2 curves were essentially parallel, suggesting a shared pathological origin, both consistent with metastases from the primary bladder tumor. ECV, extracellular volume fraction

A retrospective review of the initial postoperative spectral CT scan performed 1 month post-operation revealed no obvious sigmoid colon mass on conventional images, and the bowel wall density appeared comparable to adjacent segments (**Fig. 3A**). However, on the 50-keV VMI, the corresponding bowel segment displayed subtle but noticeable enhancement (**Fig. 3B**). The iodine-no-water image showed increased iodine uptake in this region compared with the surrounding normal bowel wall (**Fig. 3C**). On the ECV map, the same segment displayed distinctly elevated ECV values, reaching 77.9%, appearing as a patchy red region that rendered the abnormal bowel segment more distinct relative to other segments and adjacent tissues (**Fig. 3D**). The ECV was measured as described above.

**Fig. 3:**
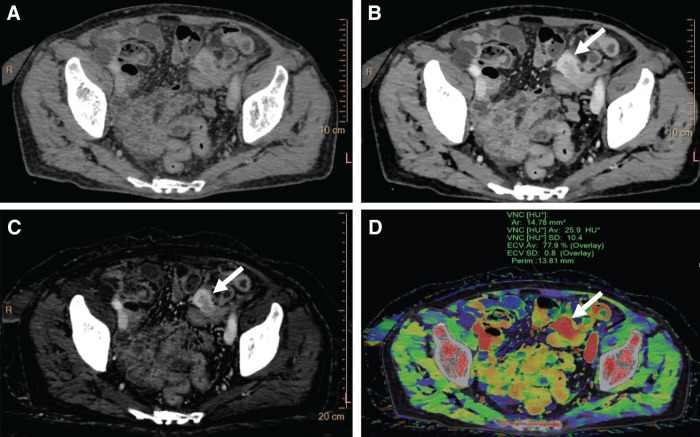
Retrospective spectral CT images 1 month post-operation. (**A**) No abnormality was observed on conventional spectral CT. (**B**) An enhanced bowel segment was visible on the 50-keV monoenergetic image (arrow). (**C**) The iodine-no-water image showed high iodine uptake in the lesion (arrow). (**D**) The ECV map demonstrated patchy red enhancement in the corresponding segment (arrow). ECV, extracellular volume fraction

## DISCUSSION

Bladder urothelial carcinoma rarely metastasizes to the GI tract. A systematic literature review was conducted using PubMed covering the period from 1983 to 2025. The following medical subject headings (MeSH) or keywords were used: (“bladder cancer” or “bladder carcinoma” or “urothelial carcinoma” or “transitional cell carcinoma” or “high grade urothelial carcinoma”) and (“metastasis” or “metastatic”) and (“rectum” or “rectal” or “sigmoid” or “descending colon” or “transverse colon” or “ascending colon” or “cecum” or “large intestine” or “large bowel” or “colon”). In addition, the “similar articles” recommended by PubMed and the reference lists of relevant studies were manually screened. Ultimately, only cases with pathologically or clinically confirmed colonic metastasis were included in the review. Only 68 cases have been reported, among which rectal involvement is relatively common (32 cases), whereas colonic metastasis is particularly rare, with only 8 cases previously reported (**Table 1**).^6–13)^ Other reported sites of involvement include the stomach and small intestine. Clinically, patients often present with nonspecific symptoms such as abdominal pain, melena, and obstruction, which may lead to misdiagnosis as primary GI malignancies or inflammatory conditions. The routes of metastasis include direct invasion, hematogenous spread, and lymphatic dissemination.^14,15)^ In earlier studies, the diagnosis of metastatic lesions primarily relied on conventional CT combined with endoscopy and pathological examination. Although cystoscopy remains essential for monitoring intravesical recurrence, it is incapable of detecting extravesical distant metastases, such as the sigmoid colon metastasis.^2,15)^ Therefore, imaging modalities that allow for the evaluation of the entire abdomen and pelvis may provide additional clinical value in identifying occult metastatic foci. In addition, conventional CT mainly depends on morphological changes, which limits its utility in diagnosing metastatic foci that have not yet caused significant morphological abnormalities.^16)^

**Table 1:** Metastases of urothelial bladder carcinoma to the colon

Author (year)	Age/sex	Metastatic site	Clinical presentation with GI involvement	Diagnostic method	Treatment after metastasis	Time to metastasis (months)	Outcome (months)
Aigen and Schapira^6)^ (1983)	66/M	Sigmoid colon, transverse colon	Constipation, weight loss, and malaise	Laparotomy disclosed, pathologic examination	Ileotransverse colostomy and loop colostomy of the descending colon	96	Not reported
Kakizawa et al.^7)^ (2006)	57/M	Sigmoid colon	Lower GI bleeding	CT, colonoscopy	Low-dose arterial vasopressin infusion via a sigmoid artery	12	Alive (5)
Blasberg et al.^8)^ (2009)	83/F	Transverse colon	Abdominal pain and obstipation	CT, pathologic examination	Right hemicolectomy	3	Not reported
Kumar et al.^9)^ (2009)	60/M	Sigmoid colon	Lower GI bleeding	Colonoscopy, biopsies	The patient refused further intervention	5	Not reported
Asfour et al.^10)^ (2014)	61/M	Colon	Intermittent constipation, diarrhea and rectal pain	CT, colonoscopy, biopsy	Palliative loop colostomy and chemoradiation	12	Died
Rohloff et al.^11)^ (2015)	49/M	Descending colon and sigmoid colon	Abdominal distention and significant weight loss	Sigmoidoscopy, barium enema, pathologic examination	Left hemicolectomy with transverse colon end colostomy	12	Alive (165)
Girão de Caires et al.^12)^ (2024)	80/M	Ileum, sigmoid colon	Diffuse abdominal pain, ileus	CT, colonoscopy, histopathological examination	Urgent laparotomy, enteroenteric anastomosis and a terminal colostomy, palliative care	Metastasis was already present at the time of primary tumor detection	Not reported
Chen et al.^13)^ (2025)	65/M	Rectum and sigmoid colon	Abdominal fullness, nausea, and changes in bowel habits	CT, colonoscopy, histopathologic examination	Patient and family declined further aggressive or invasive interventions	3	Died 3 months later
Our case (2025)	77/M	Sigmoid colon	Abdominal pain and melena	CT, colonoscopy	The patient’s family requested transfer to another institution for continued care	7	Alive (2)

F, female; GI, gastrointestinal; M, male

ECV is the combined volume fraction of the extracellular extravascular and intravascular spaces and has been shown to reflect key features of the tumor microenvironment, including microvascular density and the degree of stromal fibrosis. Malignant tumor stroma frequently exhibits pathological changes such as collagen deposition and neovascularization, which expand the extracellular space and result in elevated ECV values. Previous studies have demonstrated the efficacy of ECV in predicting lymph node metastases in thyroid and gastric carcinoma.^17,18)^ In the context of colorectal cancer, delayed-phase ECV values have been shown to increase significantly in metastatic lesions with lymphatic, neural, or vascular invasion.^18)^ However, it should be noted that elevated ECV is not specific to malignancy. Non-neoplastic conditions such as inflammation, fibrosis, edema, and vascular injury may also increase the ECV value.^19)^ In the GI tract, acute intestinal inflammation can exhibit markedly elevated ECV values due to mucosal hyperemia, capillary leakage, and submucosal edema, which may mimic or obscure neoplastic lesions.^20,21)^ Therefore, interpretation of ECV should be integrated with other imaging findings and clinical information. In our case, the elevated ECV values in the sigmoid colon, together with the spectral attenuation curve, increased suspicion for metastatic disease, while the iodine density map and the 50-keV VMI provided complementary diagnostic information.

Iodine density maps enable quantitative assessment of contrast agent uptake and are particularly useful for differentiating hypervascular tumor tissue from areas with low or absent iodine concentration.^22)^ In bladder cancer, tumor tissue has been reported to exhibit relatively high iodine concentrations.^23)^ In this particular instance, the sigmoid colon lesion exhibited a marked increase in iodine concentration, supporting its malignant nature. Similarly, low-keV VMI enhanced lesion conspicuity by amplifying iodine contrast and improving lesion-to-background differentiation. In this case, the 50-keV images substantially enhanced delineation of the sigmoid mass margins. Wang et al.^5)^ reported a case of gastric metastasis from urothelial carcinoma in which low-keV spectral images increased tumor-to-normal tissue contrast, improving lesion detection. Furthermore, spectral curves confirmed the homology between the gastric metastasis and the primary bladder lesion.

## CONCLUSIONS

Spectral CT is a multiparametric imaging technique that provides functional and quantitative information beyond conventional morphological assessment. In this case, ECV imaging revealed abnormalities in the sigmoid colon before significant structural changes were detectable on conventional CT, indicating its potential value in the early recognition of metastatic lesions. Therefore, in the follow-up of malignancies with high recurrence or a propensity for pelvic metastasis, quantitative ECV imaging with spectral CT may serve as a useful tool for the early detection of metastatic foci. Meanwhile, to the best of our knowledge, this is the first case report to demonstrate the application of spectral CT–derived ECV imaging in the early detection of sigmoid colon metastasis from bladder cancer.

## DECLARATIONS

### Availability of data and materials

The dataset supporting the conclusions of this article is included within the article.
